# Longitudinal multiple imputation approaches for body mass index or other variables with very low individual-level variability: the mibmi command in Stata

**DOI:** 10.1186/s13104-016-2365-z

**Published:** 2017-01-13

**Authors:** Evangelos Kontopantelis, Rosa Parisi, David A. Springate, David Reeves

**Affiliations:** 1 NIHR School for Primary Care Research, University of Manchester, Williamson Building, Oxford Road, Manchester, M13 9PL UK; 2 Farr Institute for Health Informatics Research, University of Manchester, Vaughan House, Portsmouth Street, Manchester, M13 9GB UK; 3 Centre for Pharmacoepidemiology & Drug Safety, University of Manchester, Stopford Building, Oxford Road, Manchester, M13 9PL UK; 4 Centre for Biostatistics, University of Manchester, JMF Building, Oxford Road, Manchester, M13 9PL UK

**Keywords:** Multiple imputation, Body mass index, Cleaning, Longitudinal data

## Abstract

**Background:**

In modern health care systems, the computerization of all aspects of clinical care has led to the development of large data repositories. For example, in the UK, large primary care databases hold millions of electronic medical records, with detailed information on diagnoses, treatments, outcomes and consultations. Careful analyses of these observational datasets of routinely collected data can complement evidence from clinical trials or even answer research questions that cannot been addressed in an experimental setting. However, ‘missingness’ is a common problem for routinely collected data, especially for biological parameters over time. Absence of complete data for the whole of a individual’s study period is a potential bias risk and standard complete-case approaches may lead to biased estimates. However, the structure of the data values makes standard cross-sectional multiple-imputation approaches unsuitable. In this paper we propose and evaluate mibmi, a new command for cleaning and imputing longitudinal body mass index data.

**Results:**

The regression-based data cleaning aspects of the algorithm can be useful when researchers analyze messy longitudinal data. Although the multiple imputation algorithm is computationally expensive, it performed similarly or even better to existing alternatives, when interpolating observations.

**Conclusion:**

The mibmi algorithm can be a useful tool for analyzing longitudinal body mass index data, or other longitudinal data with very low individual-level variability.

## Background

Missing data is a major problem for many statistical analyses, in particular for both clinical trials and routinely collected healthcare information. ‘Missingness’ is a difficult problem to address, particularly relevant to electronic medical records (EMRs), routinely collected data that can be invaluable in complementing well-designed randomized clinical trials or contributing new knowledge, especially when trials are prohibitively expensive or not possible [1, 2].

Data are generally considered to be missing under one of three possible mechanisms: missing completely at random (MCAR), missing at random (MAR) and missing not at random (MNAR). In a MCAR setting the probability of an observable data point being missing (missingness probability) does not depend on any observed or unobserved parameters. When data are MAR the missingness probability depends on observed variables, and can be accounted for by information contained in dataset. Finally, when data are MNAR the missingness probability depends on unobserved values and is very difficult to be quantified and modelled (external information is needed). In the ideal case when data are MCAR, parameter estimates are not biased in any way and the only downside of proceeding with a complete cases analysis (effectively ignoring the issue) is a loss of statistical power. This loss is not always negligible, however, especially in multiple regression analyses with many predictors where even low levels of ‘missingness’ on individual variables can result in a high total percentage of cases being dropped from analysis.

In the typical MAR scenario, the values (or categories) of a variable are associated with whether information for another variable, predictor or outcome, is missing or not. For example, under the quality and outcomes framework which is a UK primary care pay-for-performance scheme, physicians are incentivized to record the blood pressure of certain chronic condition patient groups (e.g. diabetes). Since the introduction of the scheme in 2004, annual systolic and diastolic blood measurements are almost complete in UK Primary Care Databases (Clinical Practice Research Datalink or CPRD, The Health Improvement Network or THIN, QResearch), for diabetes patients. However, data is more often missing for other patient groups, especially before 2004. Estimating the relationship between a diagnosis of diabetes and blood pressure levels is not straightforward in this context and a complete-case analysis could provide biased estimates. Currently, multiple imputation (MI) is considered the best practice to deal with this problem [3], with a possible alternative being inverse probability weighting [4]. The better performance of MI over other approaches, such as observation carried forward and complete cases, has been repeatedly confirmed [5, 6], although it is not a panacea [7]. There are ways to assess whether data are MAR [8], for example, by assessing the relationship between a predictor’s values and missingness or not in the outcome through a logistic regression. Arguably, MAR is an inaccurate term for this type of missingness and the term ‘informative missingness’ is often preferred.

In the most challenging case, data missing under a MNAR mechanism, the value of the variable that is missing is related to the reason why it is missing, and it can be a predictor or, more worryingly, an outcome. For example, body mass index (BMI) is more likely to be measured and recorded for obese patients and more likely to be missing for patients who do not look overweight. Data values that are MNAR cannot be reliably estimated from information about other variables, unless the mechanism of missingness is known, which is very uncommon. Although multiple imputation can offer some protection against MNAR mechanisms, identifying and effectively controlling for such a mechanism can very challenging [9, 10].

Multiple imputations for longitudinal data are particularly challenging, since it is necessary to account for variable correlations both within and between time points in the generation of the imputed values. Nevalainen et al. proposed an extension to cross-sectional methods for the longitudinal data setting [11], which was recently implemented in the very useful twofold algorithm for Stata [12], evaluated and found to perform well under MCAR assumptions [13]. Imputations for longitudinal sequences have been found to perform better when based on observations from each person, rather than group averages [14]. For a relatively stable over time biological parameter such as BMI, correlations with other variables within and between time points can be expected to be very small compared to BMI correlations across time points. Although models of group averages should account for these issues, we hypothesise that, specifically for BMI, there is very little information to be gained from other covariates, if they are available. Therefore we should be able to reliably impute BMI values between existing observations (interpolations) for each person, which will also give us flexibility to generate more realistic individual BMI trends rather than fluctuations around a trend mean.

To this end, we developed mibmi, a cleaning and multiple imputation algorithm for BMI or other variables with very low individual-level variability. The cleaning aspect of the algorithm identifies and sets to missing outliers that are very likely to be error values and can bias inference. The algorithm focuses on each individual to produce interpolations (between observations) and extrapolations (before first or after last observation) in a longitudinal setting for the variable of interest, provided at least two observations are available for an individual. The generated datasets are compatible with the mi family of commands in Stata.

## Methods

**Fig. 1:**
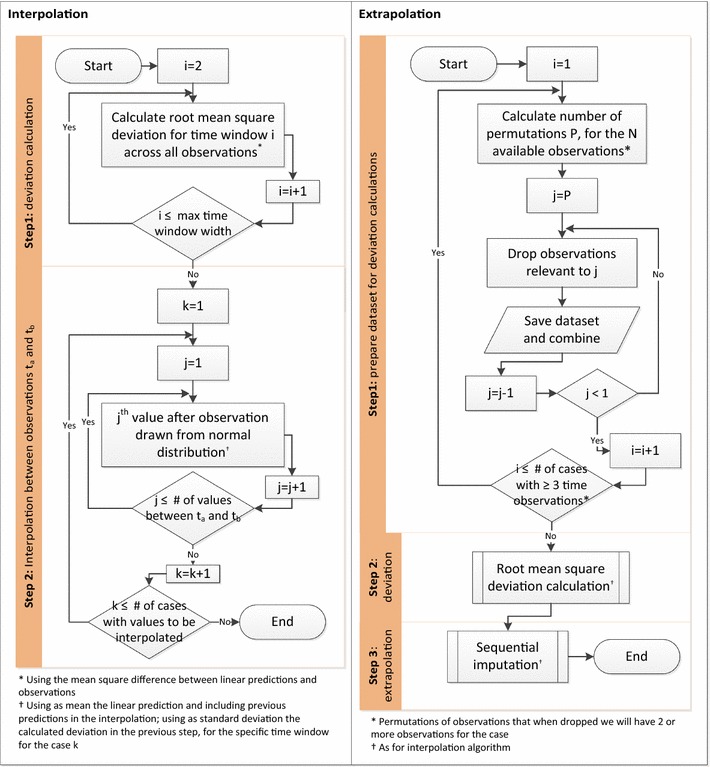
Algorithm workflow

The command includes two cleaning options. Standard cleaning limits values to a logical pre-specified range and a more advanced option uses regression-based cleaning for each individual. Provided the variable of interest is BMI and weight and height have been provided, the algorithm will use these in addition to BMI observations at all available time points, to first establish the most reliable height estimate and use that to correct BMI and/or weight values. In the standard multiple imputation setting, the command will interpolate measurements of interest for patients with at least 2 observations over the time period. Residuals are used to quantify interpolation prediction errors, for all possible time-window lengths, and these are used to introduce uncertainty in the interpolation estimates, in a multiple imputations setting. Imputed values are drawn from normal distributions, the means for which are provided by the ipolate command and the standard deviations are the standard errors for the predictions for the respective time-window length. A similar approach is used for extrapolations, if requested. The algorithm workflow for both interpolation and extrapolation is presented in Fig. 1. User defined MNAR assumptions are also allowed, under which values can be imputed through either interpolation or extrapolation. The command is computationally demanding and can take a long time to run for very large populations, especially when both interpolations and extrapolations are requested. Time-windows can be in years, months or even days, provided data completeness is reasonable. For example, in UK primary care databases, BMI is routinely recorded for people with certain chronic conditions at least once every year, since physicians are incentivized to measure it. In a clinical trial BMI may be recorded on a weekly basis and hence a much smaller time-window for analysis may be desirable.

### Cleaning

In the standard cleaning approach, the algorithm simply sets values below 8 or above 210 to missing. BMI values outside this range are extremely unlikely, across all ages [15]. If height and weight are provided, similar range restrictions are applied, between 0.81 and 2.3 m and 15–500 kgs (if age is also provided the lower limits only apply to individuals aged 10 or over). The upper and lower values threshold can be edited by the user.

Under the more advanced regression-based cleaning setting, weight and height values, when available, are used to compute a BMI score for comparison against the recorded BMI values. First, height observations are used to estimate the median height value. Since we assume height to be constant over time (unless age is provided, in which case the approach is limited to those aged 18 or over), height is replaced with the median value in all time points. Next, potentially more reliable BMI values are calculated using the ‘corrected’ height value and the available weight values (again, taking age into account if provided). As in standard cleaning, BMI values are set to missing if they are outside the [8, 210] range.

In the final step of the regression-based cleaning (and first if weight and height are not provided, for example when the variable of interest is not BMI), a linear regression of time on the variable oif interest is executed, for each individual with three or more observations. We run a separate ordinary least squares model for each individual, analogous to some extent to previously proposed random-effects modelling [16]. For time points where the ratio of absolute model residual value (observation minus prediction) over the observation is higher than 0.5 (50%), the observation is assumed to be unrealistic and is dropped. The value rejection threshold can be set by the user in the (0, 10] range.

### Interpolation

The main feature of the algorithm is imputation of missing values between observations, for each individual.Although the command and methods were originally developed for BMI imputation, they should be relevant to any variable with very low individual-level variability.

In the first step, available observations are used to quantify the error of predictions using the ipolate command. For each possible distance between time points, we assume existing observations are missing and impute them using ipolate. Subtracting each estimate from the actual observation we calculate the root mean square deviation, which we aggregate across all cases for each time-window width. Assuming a time-window width *i*, taking values between 2 (e.g. between time points 1 and 3, 2 and 4 etc) and* k*−1, if *k* is the number of time points:

1 $$\begin{aligned} {irmsd}_i=\sqrt{\frac{1}{n}\sum \limits _{j=1}^n{({pred_i}_j-{obs_i}_j)^2}} \end{aligned}$$

where *n* is the total number of cases for which a comparison is possible, across individuals and time-windows of size *i*. For example, assuming 5 time points, ${irmsd}_2$ is calculated across all patients with complete observations for time points 1–3, 2–4 and 3–5: values for time points 2, 3 and 4, respectively, are assumed to be missing and are estimated and then compared to the observed values as described by (1). In other words, the root mean square deviation is calculated pooled across all possible time windows (of a specific width) and all individuals.

The second step involves the actual imputation of missing values, using interpolation. For each individual, any observations that can be interpolated are identified. For each set of values to be imputed, between two observations in time points $t_\alpha$ and $t_\beta$, the time-window width is identified and linked to the respective root mean square deviation calculated in step 1. Next, the group of values is imputed sequentially, starting from time point $t_\alpha +1$. For $t_\alpha +1$, the value to be imputed is randomly drawn from $N({mv}_{t_\alpha +1},{irmsd}_{t_\beta -t_\alpha })$, where ${mv}_{t_\alpha +1}$ is the interpolation value provided by the ipolate command for time point $t_\alpha +1$ using $t_\alpha$ and $t_\beta$ values. The next time point for which a value is imputed is $t_\alpha +2$ (assuming $t_\beta -t_\alpha >2$), randomly drawn from $N({{mv}^{\prime}_{t_\alpha +2}},{irmsd}_{t_\beta -t_\alpha })$, where ${{mv}^{\prime}_{t_\alpha +2}}$ is the interpolation value provided by the ipolate command for time point $t_\alpha +2$ using $t_\alpha +1$ and $t_\beta$ values. In other words, for each imputed value, the immediately previous value is always taken into account, whether observed or imputed. This approach allows for imputed values that do not fluctuate unrealistically around a mean but rather simulate trends of increasing, decreasing or stable values between observations. The more imputed variables are generated, the more of these possible trends are simulated.

### Extrapolation

The algorithm will also allow missing values for an individual to be extrapolated, in a process based on the ipolate or regress commands. The extrapolation process involves three steps.

In the first step, the available dataset is edited and reshaped to allow for the comparison of predictions with observations, for all possible extrapolations. For example, if an individual’s observations are available for time points $t_\alpha$, $t_\beta$, $t_\gamma$ and $t_\delta$, the algorithm will ‘drop’ values to generate subsets on which the comparisons will take place. In this case it will generate four subsets by dropping $t_\alpha$, $t_\alpha$ and $t_\beta$, $t_\delta$, $t_\delta$ and $t_\gamma$, allowing the evaluation of what would be extrapolated values. A minimum of two observations need to be available for a subset to be of use, hence only patients with at least three observations are involved in this part of the extrapolation process. All generated sub-datasets are then combined in a single temporary file.

The temporary file is then used to calculate root mean square deviation estimates, in a similar way as for interpolation, but in this case they are much larger (since the methods we use to empirically quantify deviation are less accurate). Users can choose either an ipolate or a computationally more expensive regress based estimation for all the values that were ‘dropped’ in the previous step, with the former using the closest two and the latter using all available observations. For each possible distance *i* between the ‘dropped’ value to be imputed and the closest observation, the root mean square deviation is estimated using (1). In this case, however, we call it ${ermsd}_i$ with *n* in the formula being the total number of cases in the temporary file, for which a comparison is possible for time distance *i*.

In the last step, for each individual, the missing values that can be estimated using extrapolation are identified and linked to the respective root mean square deviation calculated in the previous step. As with interpolation, extrapolation values are imputed sequentially for each individual, starting from the time point closest to an observation. Assuming an observation exists for time point $t_\alpha$ and an extrapolation can be calculated for $t_\alpha +1$, the value to be imputed will be randomly drawn from $N({mv}_{t_\alpha +1},{ermsd}_1)$, where ${mv}_{t_\alpha +1}$ is the extrapolation value provided by ipolate or regress for time point $t_\alpha +1$. Assuming $t_\alpha +2$ can be extrapolated, it is randomly drawn from $N(\mathop {mv_{{t_{\alpha } + 2}} }\limits^{{\prime }} ,ermsd_{2} )$, where $\mathop {mv_{{t_{\alpha } + 2}} }\limits^{\prime }$ the extrapolation value provided by ipolate or regress for time point $t_\alpha +2$, but including the imputed value for $t_\alpha +1$ in the process. The algorithm continues sequentially and imputes values for all time points where an extrapolations is possible, for each individual, simulating realistic variable trends (as many for each individual as the number of variables to be generated in the imputation process). Draws for both interpolation and extrapolation are effectively constrained to acceptable values in the [8, 210] range, although in our experience this constraint should never have to be invoked for interpolations and only very rarely for extrapolations. It should also be noted that each drawn interpolation or extrapolation is assumed to be exact, within the specific dataset, and only through a multiple imputation process will the uncertainty in the estimate be fully captured.

## The mibmi command

### Syntax


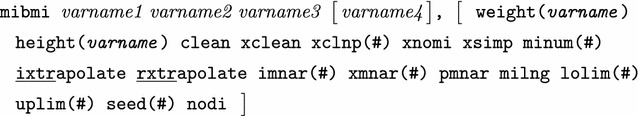


### Variables

The command requires three variables to be provided, in the following order: the unique within time individual identifier (*varname1*); a linear time variable to define monthly, yearly or other time-windows (*varname2*); and the main variable of interest, usually the BMI (*varname3*). An optional variable with the age in years can also be provided (*varname4*), which is used in the simple cleaning process, if requested. Also note that the data needs to be in long rather than wide format, in relation to time. A backup variable for the original variable of interest is created in *_varname3*.

### Options

#### Cleaning

weight(*varname*) Weight in kilograms. If provided along with height, both variables will be used to correct BMI and/or height and weight observations. Only relevant for BMI imputation.

height(*varname*) Height in metres. If provided along with weight, both variables will be used to correct BMI and/or height and weight observations. Only relevant for BMI imputation.

clean Standard cleaning option requested to set unrealistic values to missing (default is >210 or <8). Assuming the variable of interest is BMI, if weight and height have been provided they are also cleaned at this stage, taking age into account if it has been provided.

xclean More advanced cleaning option that uses regression modelling to identify unrealistic changes in the variable of interest, which are very likely input errors, and set them to missing. If BMI is the variable of interest, provided weight and height values will be taken into account: first, weight, height and BMI values are investigated longitudinally to try to verify the subject’s height (accounting for age, if provided). Then, using this ’most likely’ height value, BMI values are corrected if needed. The second stage, which is the only stage if the variable of interest is not BMI, involves running a regression model for each subject to identify unrealistic changes in BMI and set them to missing. The threshold over which the observations are set to missing is set with the xclnp(#) option.

xclnp(#) Threshold for regression cleaning, defined as absolute residual value (i.e. observed minus prediction) over observed value. The default value is 0.5 (i.e. 50%).

xnomi By default the command is a multiple imputation command. This option suppresses multiple imputations and hence allows the command to be used solely for cleaning.

xsimp By default the command is a multiple imputation command. This option suppresses multiple imputations and allows simple imputation, with no standard errors calculated and implemented in either intrapolations or extrapolations. It can be issued with the ixtrapolate or rxtrapolate options

#### Multiple imputation

minum(#) Number of multiple imputations. The default is five.

ixtr apolate Requests extrapolation (in addition to interpolation), using the ipolate command. Standard errors for ipolate predictions are calculated (for various time-windows), by removing observed BMI values and calculating model performance for them. The ipolate command (with the extrapolation option) is then used to sequentially impute extrapolated values: starting from the time points closest to the observed values and moving further away. At each stage, values are drawn from a normal distribution the mean for which is provided by the ipolate command and its standard deviation is the standard error for the predictions for the respective time-window.

rxtr apolate Requests extrapolation (in addition to interpolation), using the regress command. Standard errors for regress predictions are calculated (for various time-windows), by removing observed BMI values and calculating model performance for them. The regress command is then used to sequentially impute extrapolated values: starting from the time points closest to the observed values and moving further away. At each stage, values are drawn from a normal distribution the mean for which is provided by the ipolate command and its standard deviation is the standard error for the predictions for the respective time-window.

imnar(#) Missing not at random (MNAR) assumption for interpolated values. Increases or decreases the predictions by the value specified, in the $[-50,+50]$ range but within the logical range for BMI.

xmnar(#) Missing not at random (MNAR) assumption for extrapolated values. Increases or decreases the predictions by the value specified, in the $[-50,+50]$ range but within the logical range for BMI.

pmnar Indicates that a percentage change, rather than an absolute value increase/decrease, is to be used for the MNAR mechanism(s). If this option is specified, options imnar(#) and xmnar(#) will accept values in the [−0.9, +0.9] range, indicating a percentage change between −90 and 90%. Users should be aware that increases and decreases are not symmetrical under this option.

milng Requests the multiple imputations dataset in mlong format instead of wide, the default.

#### Other

lolim(#) Lower value threshold below which observations are dropped when using option clean and imputations are constrained. The default value, for adult BMI, is set to 8.

uplim(#) Upper value threshold above which observations are dropped when using clean and imputations are constrained. The default value, for adult BMI, is set to 210.

seed(#) Set initial value of random-number seed, for the simulations. The default is 7. See set seed.

nodi Do not display progress. Not recommended since imputation can take a very long time for large databases.

### Saved results

The mibmi command does not return any scalars but an edited dataset, mi compatible if imputations are performed. In that case, additional variables are included. The mi standard variable *_mi_miss* includes binary information on whether values are missing or not. Variables *_mi_ipat* and *_mi_xpat* flag patients for which at least one value has been interpolated or extrapolated, respectively (the latter is only present if extrapolations have been requested). Assuming the default mi wide format is used, imputed variables are available in the usual Stata format *_i_varname3*, including observed and imputed values (the number of variables is defined by minum(#)). Finally, *_i_iinfo* and *_i_xinfo*, if extrapolations are requested, include information on the imputed observations and the validity of the imputed values for the respective variable, i.e. they flag whether the imputation process would have provided a value outside the pre-defined logical range and had to be corrected by setting to the minimum or maximum allowed. Such a scenario is extremely unlikely for interpolations and *_i_iinfo* variables do not really vary (zero for all imputed values, missing for observations). However, it does happen for extrapolations, although rarely, and on occasion the *_i_xinfo* variables include non-zero values. This seems to be more likely with the default and faster ipolate approach, which only accounts for two observations during the prediction process and is more sensitive to extreme or incorrect values.

### Example

We explore the mibmi command with an anonymized sub-sample of diabetes patients from the Clinical Practice Research Datalink (CPRD). The algorithm was used on the full sample, in a recent investigation of the relationship between biological variables and mortality [17]. Here we present a significantly reduced sub-sample, edited using random processes to overcome sharing restrictions. The dataset holds information on age (in years), mean weight (in kg), height (in metres), mean BMI and the number of different drugs prescribed, from 1 April 2004 to 31 March 2012, aggregated into eight financial years (1 April to 31 March). In this series of examples we demonstrate the use of mibmi in cleaning and imputing BMI data, before using multi-level Poisson regression modelling to quantify the association between BMI and the number of drug prescription over an 1-year period (in either simple analyses or a multiple imputation framework).
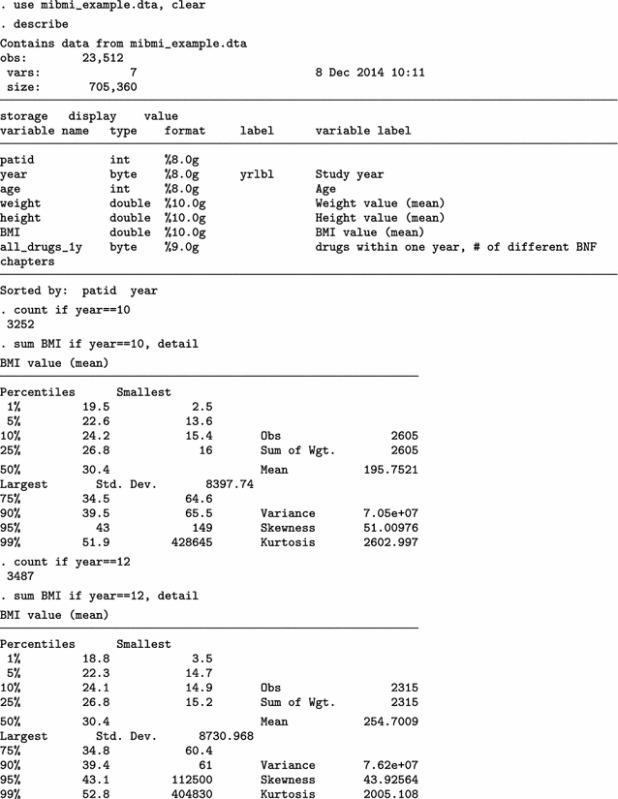


We present BMI characteristics for two representative time points: 2009/10 (year 10) and 2011/12 (year 12), the last year of the study. At least one BMI measurement is available for 2605 of 3252 eligible individuals in 2009/10 (80.1%) and for 2315 of 3487 in 2011/12 (66.4%). A few very high BMI values are obviously erroneous. Nevertheless, we make no corrections and proceed to investigate the relationship between average BMI and polypharmacy, using a multi-level Poisson regression model.
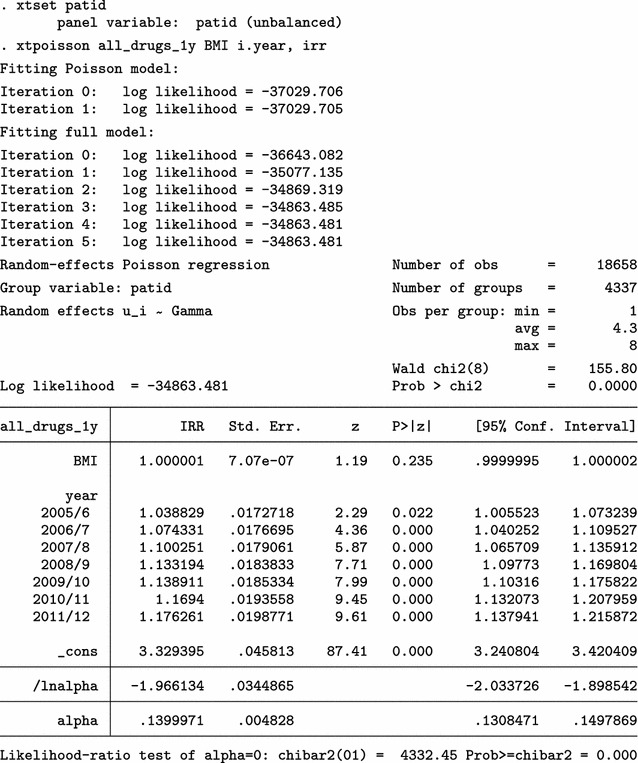


The analysis on the original dataset indicates that the relationship between BMI and polypharmacy is very weak and non-significant. Next, we only use the simple cleaning approach of the mibmi command to remove unrealistic BMI values and correct using the provided weight and height, if possible.
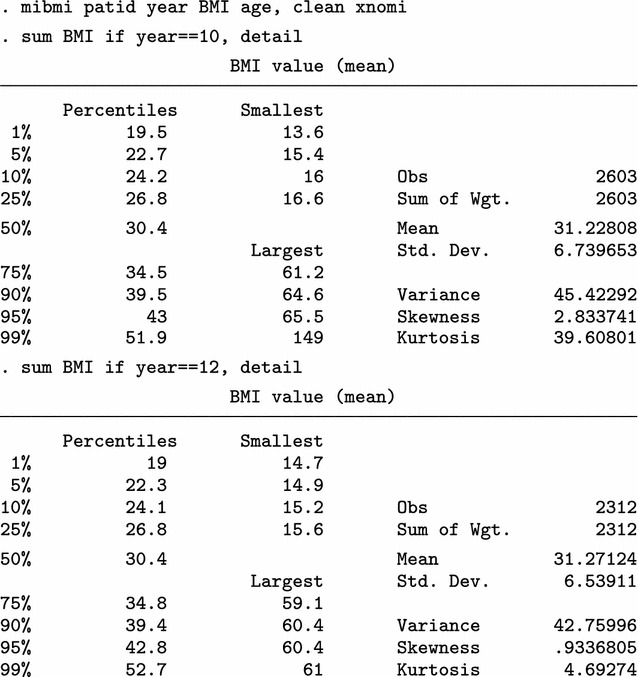


A handful of extreme BMI observations were set to missing but further corrections have been performed, based on available weight and height measurements. We repeat the multi-level Poisson regression analysis on this cleaned dataset.
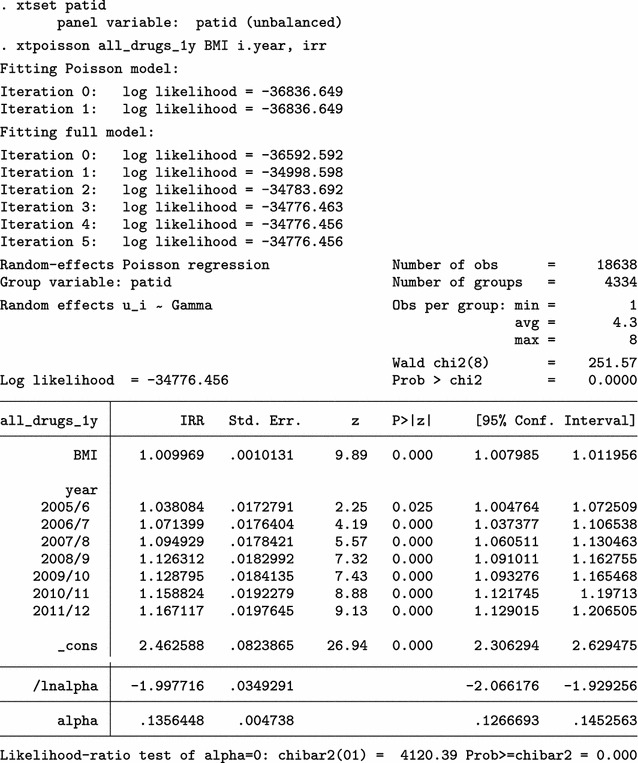


Analysis on the (simply) cleaned datasets suggests there is statistically significant relationship between BMI and polypharmacy. Next, we go one step further with the mibmi command by requesting simple and advanced cleaning on the original dataset.
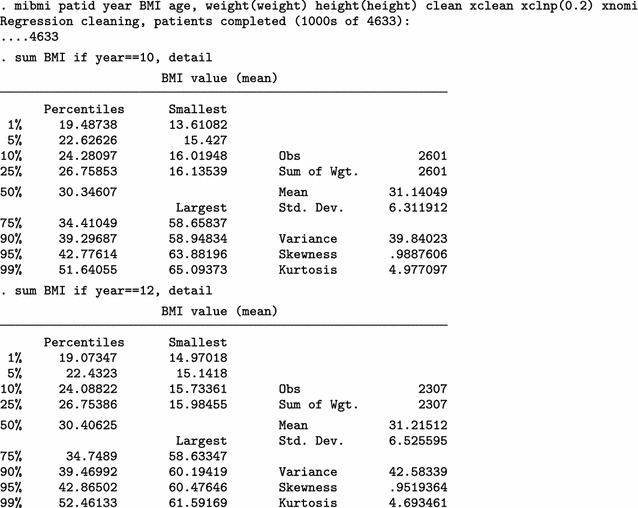


A few more values are dropped due to regression cleaning (with a low 20% threshold defined by the xclnp(#) option). Repeating the analysis, we obtain similar results.
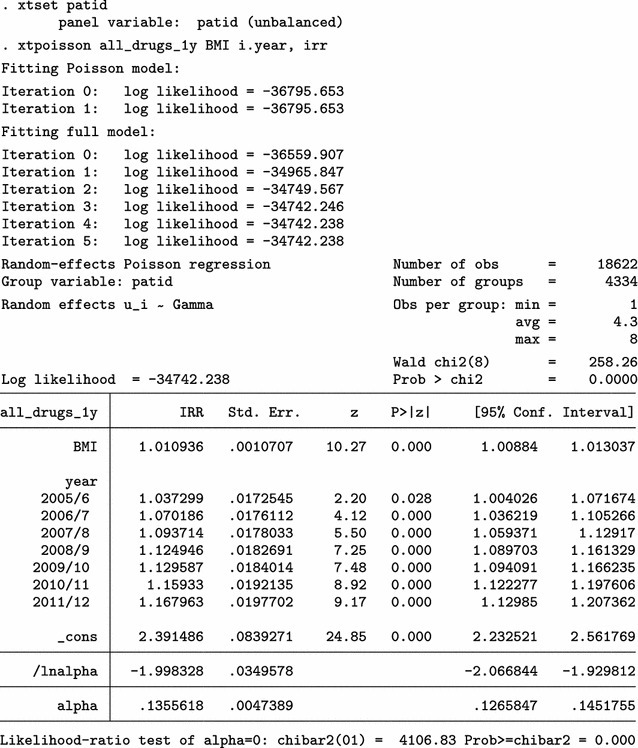


Next, we use mibmi not only to clean the data but also to generate a set of three MI variables holding imputed values.
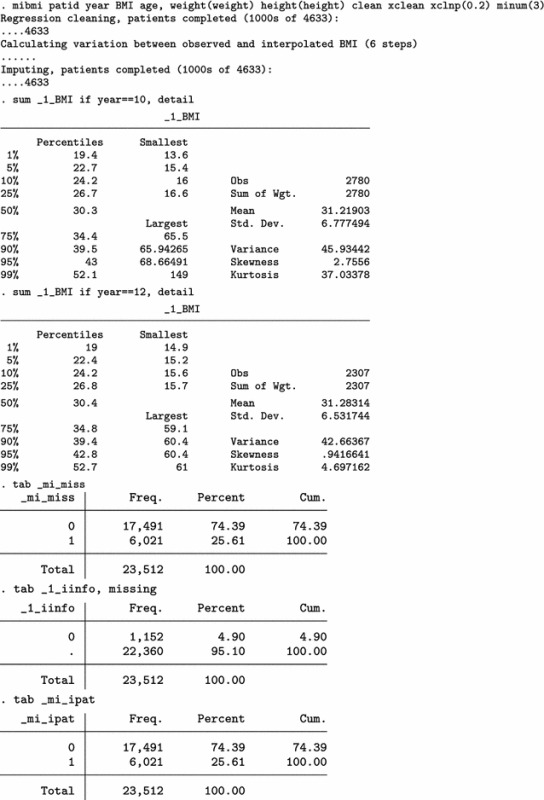


We focus on the characteristics of variable _1_BMI, but the imputed cases (not imputed values) are identical across all three variables. For 2009/10 (year 10) and each imputation set, the algorithm imputed 179 observations (2780 now, compared to 2601 when only using simple and advanced cleaning). Unsurprisingly, no values are interpolated for the last time point, 20111/12 (year 12). Three new variables provide information on the interpolation process: _mi_miss flags all missing BMI observations; _1_iinfo flags cases where interpolated values for _1_BMI were unrealistic and had to be constrained (in this example there were none, amongst the 1152 that were imputed); and _mi_ipat flags all patients for whom at least one observation was interpolated, at any point in time. The role of _mi_ipat is to allow users to easily obtain the number of patients with at least one interpolation:
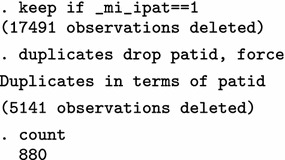


Using this interpolation dataset to run multiple imputation analyses, with the mi estimate prefix, we obtain similar results.
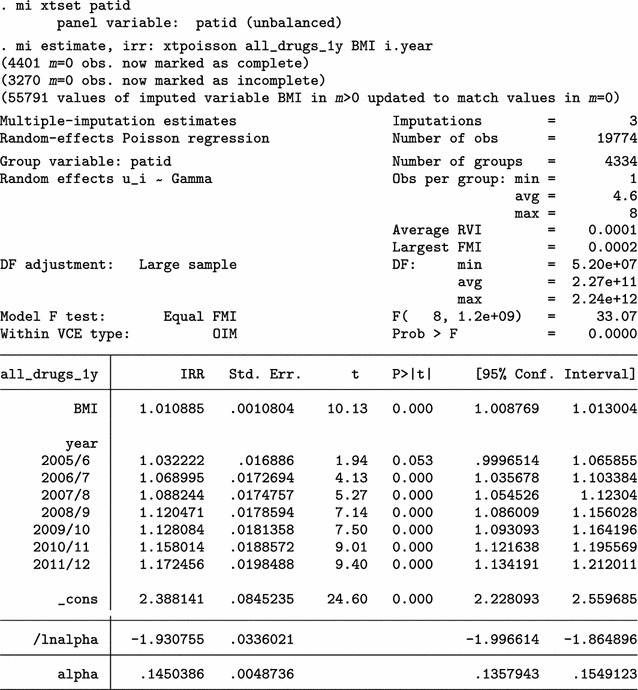


Finally, we can use all four aspects of mibmi with the original dataset: simple and advanced cleaning, interpolation and extrapolation.
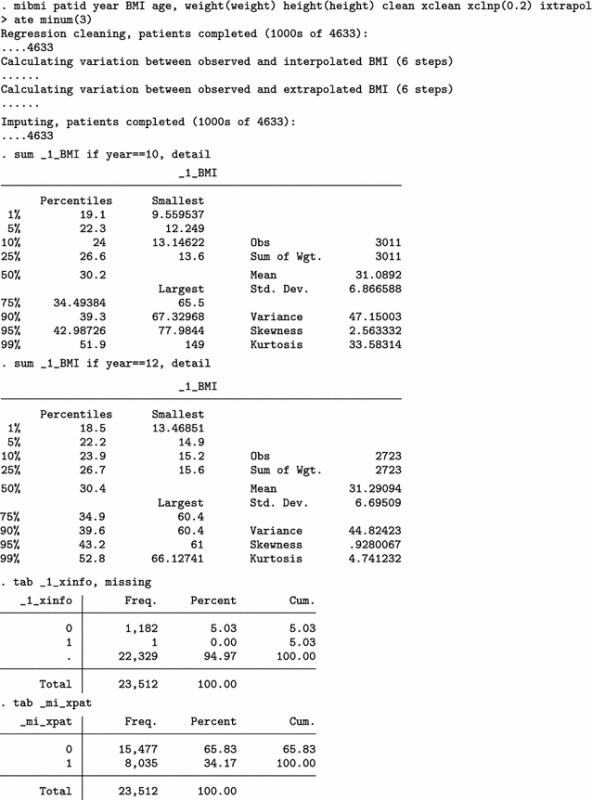


Again, we focus on the characteristics of variable _1_BMI. For 2009/10 (year 10) and each imputation set, the algorithm now imputed 410 observations, of which 213 are extrapolations (3011 now, compared to 2780 with interpolation and cleaning and 2601 with cleaning only). For the last time point, 20111/12 (year 12), 416 values were imputed with extrapolation (2723 now, compared to 2307 before) . Additional new variables provide information on the extrapolation process: _1_xinfo flags cases where interpolated values for _1_BMI were unrealistic and had to be constrained (in this example there was one amongst the 1183 extrapolated values); and _mi_xpat flags all patients for whom at least one observation was interpolated, at any point in time (with a role similar to _mi_ipat, allowing users to obtain the number of patients with at least one extrapolation).

Results from a multiple imputation analysis on the final dataset obtained with mibmi were similar to those previously obtained, as expected. Practically, the requested imputations are assuming MCAR missingness since there is no conditional missingness on observed data, and hence inference estimates should be very similar to what we observed previously. However, this is not necessarily the case for standard errors (although in this example they are):
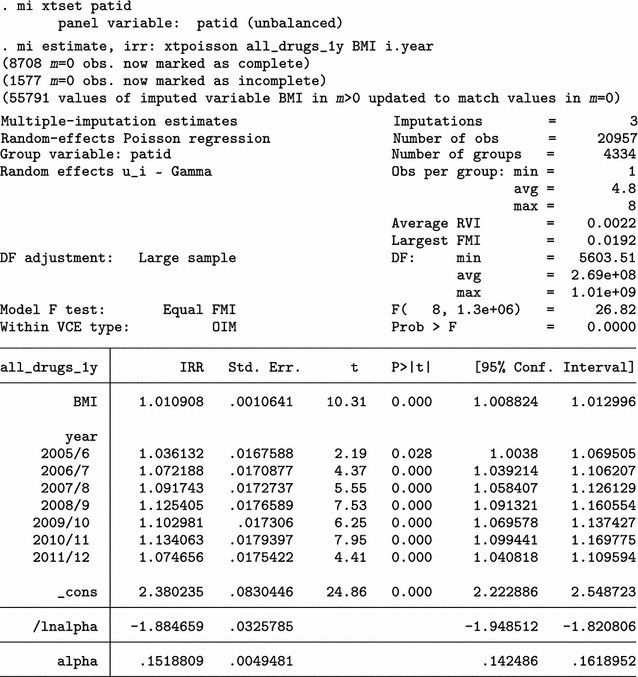


### Performance

To assess the performance of mibmi, in relation to the recently presented twofold algorithm, we used a version of the diabetes patients dataset we presented previously. For this exercise, the dataset included additional information on HbA1c (glucose), systolic and diastolic blood pressure and total cholesterol. First, we applied the mibmi algorithm with the simple and regression cleaning options to obtain a more reliable measure for BMI, thus not allowing extreme and erroneous values to affect the comparison.Then we performed two assessments of performance, when one or three values were missing between two observations for each individual. We did not choose to evaluate through a simulations framework since the assumptions under which we would have simulated the data would be critical to the analyses and the evaluation could be seen as self-fulfilling prophecy. Rather, we used real data to assess deviations from observations. Therefore, we could not evaluate the performance (e.g. coverage, power) of the inferential models since the true effects and associations were unknown.

In the first assessment, we randomly selected 10,000 people with 3 or more BMI measurements over the study period and we randomly set one BMI observation per person to missing. We then used mibmi, with both simple (×1) and multiple imputation options (×100), and twofold in which we used all five available biological parameters for the multiple imputations. Under a multiple imputations approach, we obtained 100 BMI variables with imputed values, for each algorithm. Each set was then aggregated and we obtained their mean value for each of the 10,000 ‘missing’ observations. Finally, these aggregates, as well as the simple imputation BMI from mibmi, were compared to the ‘true’ BMI values to calculate absolute mean differences (mean error). Table 1 presents the overall results and for interpolated and extrapolated values separately, since the underlying principles in their imputations are different, for mibmi at least. Performance for interpolated values appears to be similar while twofold performs better for extrapolations.

**Table 1:** Mean errors between observed and imputed BMI values, one missing value per individual

Cases	Method^a^	Obs.	Mean	Std.Dev	Min	Max
All^b^	Simple ×1	10000	1.113	1.353	0.000	26.900
	mibmi ×100	10000	1.120	1.351	0.000	26.888
	Twofold ×100	10000	0.949	1.026	0.000	15.481
Interpolation	Simple ×1	6132	0.801	0.819	0.000	11.651
	mibmi ×100	6132	0.808	0.819	0.001	11.429
	Twofold ×100	6132	0.804	0.810	0.000	11.180
Extrapolation	Simple ×1	3868	1.606	1.808	0.000	26.900
	mibmi ×100	3868	1.614	1.805	0.000	26.888
	Twofold ×100	3868	1.179	1.260	0.001	15.481

^a^Simple refers to a single imputation that ignores variability in the observations (option xsimp); mibmi refers to the default multiple imputation approach with the command and 100 imputations; twofold refers to the twofold algorithm described in the paper and 100 imputations

^b^All refers to both interpolations (between observations imputations) and extrapolations (not between observations imputations)

**Fig. 2:**
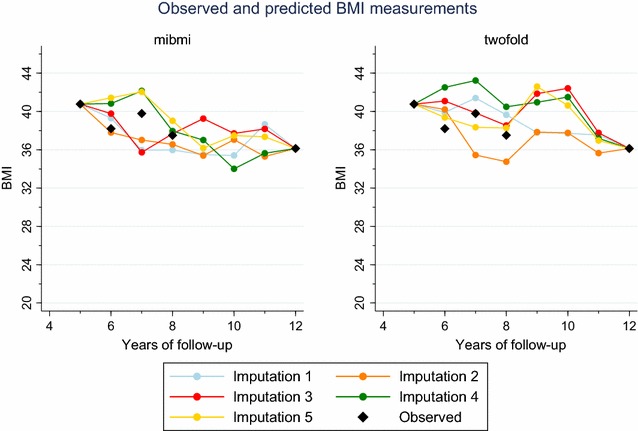
Predictions example for a single patient

In the second assessment, which focused on interpolation, we again randomly selected 10,000 people but this time with 5 or more BMI measurements over the study period. Next, for each individual, we randomly set three concurrent BMI observations to missing but ensuring these were observations that would be imputed as interpolations under the mibmi algorithm (i.e. observations were available both before and after these ‘missing’ values, for all patients). As before, we used mibmi, with both simple and multiple imputation options, and twofold with the five available biological parameters for the multiple imputations and we aggregated to obtain mean errors. Results are presented in Table 2, both overall and for each of the three sequential observations that we set to missing. Performance was better with mibmi, especially for the second time point, the one furthest away from observations. A prediction example using a single patient is presented in Fig. 2.

**Table 2:** Mean errors between observed and imputed BMI values, three sequential missing values per individual (interpolation only)

Cases	Method^a^	Obs.	Mean	Std.Dev	Min	Max
All^b^	Simple ×1	30,000	0.980	1.002	0.000	16.017
	mibmi ×100	30,000	0.989	1.004	0.000	16.034
	Twofold ×100	30,000	1.137	1.155	0.000	18.318
Time point 1	Simple ×1	10,000	0.935	0.945	0.000	9.829
	mibmi ×100	10,000	0.943	0.947	0.000	9.779
	Twofold ×100	10,000	1.094	1.114	0.000	18.318
Time point 2	Simple ×1	10,000	1.059	1.068	0.000	16.017
	mibmi ×100	10,000	1.069	1.071	0.000	16.034
	Twofold ×100	10,000	1.234	1.231	0.000	17.126
Time point 3	Simple ×1	10,000	0.947	0.984	0.000	10.645
	mibmi ×100	10,000	0.955	0.985	0.000	10.538
	Twofold ×100	10,000	1.084	1.111	0.000	13.473

^a^Simple refers to a single imputation that ignores variability in the observations (option xsimp); mibmi refers to the default multiple imputation approach with the command and 100 imputations; twofold refers to the twofold algorithm described in the paper and 100 imputations

^b^All refers to aggregates across all three time points

These results indicate that, for interpolating BMI values, there is little useful information in other biological parameters and the additional effort of obtaining them is not justified. The mibmi algorithm generates realistic linear or curvilinear trends for BMI over time and the higher computational complexity pays off more as the number of concurrent missing values increases. However, for extrapolating BMI values, performance is better with the twofold fully conditional specification algorithm and use of all biological parameters, at least when only two observations per individual are available. In such a scenario, each extrapolation is based on an individual-level model that uses only two observations which, unsurprisingly, can generate extreme values in some cases. Although the accuracy of extrapolation predictions might improve for mibmi as the number of available observations increases, performance with the twofold algorithm should remain better.

## Discussion

In this paper we presented mibmi, a new command for cleaning and imputing BMI values, or other variables with very low individual-level variability, in longitudinal settings. Using a pseudo-anonymised dataset from the Clinical Practice Research Datalink we described the command’s cleaning and imputation functions over a few examples and we also assessed its performance. The command is available to download from the ssc archive by typing *ssc install mibmi* within Stata. Alternatively readers can automatically download from the first author’s personal web page by typing *net from* http://statanalysis.co.uk within Stata and following the instructions.

We argue that mibmi can be a useful tool for researchers who wish to use longitudinal values for BMI or other variables with very low individual-level variability, in descriptive or inferential analyses. The command is fully compatible with the mi family of Stata and we incorporated numerous features to allow for flexibility in the imputation process, allowing the user to assume certain MNAR mechanisms. The same processes could be used for imputation of other parameters, providing one can assume very strong correlation over time and linearity.

The algorithm’s advantage is its ability to provide multiple datasets with imputed values for the variable of interest when no other information is available, except for an individual identifier and time. For interpolations, BMI performance was overall better than in other multiple imputation approaches that use additional biological data. Because of the command’s individual by individual approach, the interpolation and, especially, extrapolation processes are computationally expensive and, for very large datasets (of hundreds of thousands of patients), the command can take weeks to execute. When multiple imputation is selected, we recommend 5 generated datasets. However, the process can be parallelized and for large centralized data repositories, like the UK Primary Care Databases (CPRD, THIN, QResearch), mibmi could be applied once at a high level and the imputed BMI values distributed to users when requested, on a protocol-by-protocol basis. The algorithm will effectively ignore patients with fewer than 2 BMI values over time and hence researchers are unlikely to have a complete final dataset to analyze. Also note that users who extrapolate should take care to impute at appropriate times only (e.g. not when age <18).

In the context of BMI imputation, when additional biological information is available (e.g. blood pressure values), we advise its use in conjunction with twofold, especially for extrapolations. In the first step, users can execute mibmi to obtain a more reliable BMI variable through the cleaning options and generate interpolated values for patients with at least two observations over the study period. In the second step they can use the generated variable with the twofold algorithm, to obtain multiple imputations for BMI and other variables.

## Abbreviations

BMI: body mass index
CPRD: Clinical Practice Research Datalink
EMR: electronic medical records
MAR: missing at random
MCAR: missing completely at random
MNAR: missing not at random
THIN: the health improvement network
UK: United Kingdom
